# Supplementary material for: An open-source framework for large-scale, flexible evaluation of biomedical text mining systems
Source: J Biomed Discov Collab. 2008 Jan 29;3:1. doi: 10.1186/1747-5333-3-1 (PMC2276192; doi:10.1186/1747-5333-3-1)
Supplement: Additional file 1 — Evaluation platform source code. The additional file contains the software evaluation framework discussed in this paper. The most current release can be downloaded from SourceForge.net [1]. See the accompanying README file for instructions on installation and use. Also included in the distribution is a collection of UIMA wrappers for some commonly used BioNLP tools and annotated corpora. [file 1747-5333-3-1-S1.zip › bionlp_uima_repository-1.0.1/docs/api/src-html/edu/uchsc/ccp/util/external/kex/KeXEISystem.html]

```
001    /*
002     * Created on Aug 15, 2005
003     *
004     *  Changes:
005     *      03/23/06 - wab - General refactoring- removing duplicated code in all EISystems, cleaning up span detection algorithm
006     */
007    package edu.uchsc.ccp.util.external.kex;
008    
009    import java.io.BufferedOutputStream;
010    import java.io.BufferedReader;
011    import java.io.File;
012    import java.io.FileInputStream;
013    import java.io.FileOutputStream;
014    import java.io.IOException;
015    import java.io.InputStreamReader;
016    import java.io.PrintStream;
017    import java.util.ArrayList;
018    import java.util.List;
019    import java.util.regex.Matcher;
020    import java.util.regex.Pattern;
021    
022    import edu.uchsc.ccp.util.RunExecutable;
023    import edu.uchsc.ccp.util.nlp.GenericTextUtils;
024    import edu.uchsc.ccp.util.nlp.annotation.Annotator;
025    import edu.uchsc.ccp.util.nlp.annotation.TextAnnotation;
026    import edu.uchsc.ccp.util.nlp.ei.EIFileBasedSystem;
027    import edu.uchsc.ccp.util.nlp.mention.ClassMentionTypes;
028    
029    /**
030     * 
031     * @author williamb
032     * 
033     * NOTE: this will only run in a LINUX/UNIX environment due to LINUX specific commands in KeXEISystem.createExecFile
034     */
035    
036    public class KeXEISystem implements EIFileBasedSystem {
037        private final boolean DEBUG = false;
038    
039        private String baseDirectory;
040    
041        private String workDirectory;
042    
043        private final String kexOutputFilename = "KeX_output.txt";
044    
045        private final String execFilename = "kex_exec.sh";
046    
047        private final String inputFilename = "kex_input.txt"; // input file used for acceptStringInput
048    
049        /**
050         * Initialize a new KeX EI System. <br>
051         * NOTE: this will only run in a LINUX/UNIX environment due to LINUX specific commands in KeXEISystem.createExecFile
052         * 
053         * @param baseDirectory
054         *            the absolute path to the directory where the bin/ directory is located; there MUST also exist a baseDirectory/work
055         *            directory
056         */
057        public KeXEISystem(String baseDirectory) {
058            /* Initialize the base and work directories */
059            this.baseDirectory = baseDirectory;
060            this.workDirectory = baseDirectory + "/work";
061            
062            createExecFile(workDirectory + "/" + inputFilename);
063            
064            /* make sure any previous output files are no longer there*/
065            this.reset();
066        }
067    
068        /*
069         * Create the script to run KeX. This script incorporates the specific environmental variables that must be set before calling "exprot"
070         */
071        private void createExecFile(String inputFilename) {
072            try {
073                PrintStream execFile = new PrintStream(new BufferedOutputStream(new FileOutputStream(baseDirectory + "/" + execFilename)));
074    
075                /* Delete the old script file if it exists */
076                File f = new File(workDirectory + "/" + kexOutputFilename);
077                if (f.exists()) {
078                    f.delete();
079                }
080    
081                /* KeX requires a system variable: KEX to be set */
082                execFile.println("export KEX=" + baseDirectory + ";");
083    
084                /* KeX requires . to be in the path */
085                execFile.println("export PATH=$PATH:.;");
086    
087                /* change to the bin directory */
088                execFile.println("cd " + baseDirectory + "/bin;");
089    
090                /* execute KeX */
091                execFile.println("./exprot -p " + inputFilename + " " + workDirectory + "/" + kexOutputFilename + ";");
092    
093                execFile.close();
094    
095                // make execFile executable on the local file system
096                RunExecutable.run("chmod 755 " + baseDirectory + "/" + execFilename, false);
097    
098            } catch (IOException ioe) {
099                System.err.println("IOException while creating kex_exec.sh file in KeXEISystem...");
100                ioe.printStackTrace();
101            }
102        }
103    
104        /**
105         * Return the outputFileName and path
106         */
107        public String getOutputFilename() {
108            return workDirectory + "/" + kexOutputFilename;
109        }
110    
111        /**
112         * Deletes the previous output file if it exists. This is necessary because KeX refuses to overwrite an existing output file.
113         */
114        public void reset() {
115            /* delete the output file, KeX will not overwrite it */
116            File f = new File(workDirectory + "/" + kexOutputFilename);
117            f.delete();
118        }
119    
120        /**
121         * Works as it would when using KeX from the command line. The input argument is a file where each line represents an individual
122         * document to be tagged with gene names. Each line must take the form of:<br>
123         * 
124         * @@ID DocumentText
125         */
126        public List<TextAnnotation> getEntitiesFromFile(String inputFile) {
127            /* generate the script file */
128             createExecFile(inputFile);
129             
130            /* run KeX to generate the output file */
131            runKeX();
132            
133            return parseEntitiesFromFile(inputFile);
134        }
135    
136        /*
137         * Run KeX on the input file, and generate the output file
138         */
139        private void runKeX() {
140            if (DEBUG) {
141                System.out.print("running KeX...");
142                RunExecutable.run(baseDirectory + "/" + execFilename, true);
143                System.out.println("complete.");
144            } else {
145                RunExecutable.run(baseDirectory + "/" + execFilename, false);
146            }
147        }
148    
149        /**
150         * Take a String as input, create a temporary input file, then run KeX just as you would from the command line. The document ID is
151         * appended to the input string in order to create the expected input format.
152         */
153        public List<TextAnnotation> getEntitiesFromString(String originalText, String originalDocumentID) {
154            /* Create a temporary input file */
155            PrintStream ps = null;
156            try {
157                ps = GenericTextUtils.openPrintStream(workDirectory + "/" + inputFilename);
158            } catch (IOException ioe) {
159                ioe.printStackTrace();
160            }
161            /* Write the document ID followed by the document text */
162            ps.println("@@" + originalDocumentID);
163            ps.println(originalText);
164    
165            /* Save the temporary input file */
166            ps.close();
167    
168            /* Run KeX to generate the output file */
169            runKeX();
170            /*
171             * We could delete the temporary input file here, but since it will generally be relatively small, and KeX will often be called
172             * repeatedly, we have chosen to just overwrite the temporary file each time, thereby conserving the time that would be needed to
173             * delete it after each execution.
174             */
175            List<TextAnnotation> annotationsToReturn = parseEntitiesFromString(originalText, originalDocumentID);
176            this.reset();
177            return annotationsToReturn;
178            
179        }
180    
181        /*
182         * get protein annotations from the original text
183         */
184        private List<TextAnnotation> parseHitsFromKeXOutputFile(String taggedDocumentID, String taggedText, String originalDocumentID,
185                String originalText) {
186            List<TextAnnotation> returnHits = new ArrayList<TextAnnotation>();
187            try {
188                /* Initialize the Regex that will be used for finding the text thought to be protein names */
189                Pattern genePattern = Pattern.compile("<p>(.*?)</p>");
190                Matcher geneMatcher;
191    
192                if (DEBUG) {
193                    System.out.println("TAGGED TEXT: [" + taggedDocumentID + "] " + taggedText);
194                    System.out.println("ORIGINAL TEXT: [" + originalDocumentID + "] " + originalText);
195                }
196    
197                /*
198                 * Saftey check: make sure the document IDs of the tagged text and the original text match before continuing; if they do not,
199                 * then error.
200                 */
201                if (originalDocumentID.equals(taggedDocumentID)) {
202                    /* Find all of the tagged protein names by searching for <p>protein names inside</p> in the KeX output */
203                    geneMatcher = genePattern.matcher(taggedText);
204                    String geneText = "";
205                    
206                    /*
207                     * In order to ensure we capture duplicate mentions of the same protein name in the text, we must keep track of where we start
208                     * to search for matches in the text. If returnHits is empty, then start looking from zero, else start looking from the end
209                     * index of the last annotation added to the returnHits list
210                     */
211                    int startLookingForMatchesAtIndex = 0;
212                    
213                    /* Initialize an annotator */
214                    Annotator kexAnnotator = new Annotator(new Integer(3), "", "KeX", "CBRC");
215                    
216                    while (geneMatcher.find()) {
217                        geneText = geneMatcher.group(1);
218                        if (DEBUG) {
219                            System.out.println("Found a protein... text= " + geneText + " start_index= " + geneMatcher.start());
220                        }
221    
222                        /* Find all instances of the found protein text within the original text */
223                        List<TextAnnotation> proteinAnnotations = GenericTextUtils.findTargetInText(geneText, originalText, startLookingForMatchesAtIndex ,originalDocumentID, ClassMentionTypes.PROTEIN, kexAnnotator, false);
224                        
225                        /* and add them to the list to return. */
226                        returnHits.addAll(proteinAnnotations);
227                        
228                        if (returnHits.size() == 0) {
229                            System.out.println("***********  KeX missed gene: " + geneText + "  *********** + documentID: " + originalDocumentID + " startIndex" + geneMatcher.start());
230                        }
231                        
232                        /* Update startLookingForMatchesAtIndex to be the end of the span of the most recent additiong to the returnHits lise. */
233                        if (returnHits.size() > 0) {
234                            startLookingForMatchesAtIndex = returnHits.get(returnHits.size()-1).getAnnotationSpanEnd();
235                        }
236                    }
237                } else { /* the original text and tagged text document IDs to do match, so there must be a problem somewhere. */
238                    System.err.println("ERROR, document IDs do not match up: tagged_docID: " + taggedDocumentID + "  orig_docID: "
239                            + originalDocumentID);
240                }
241    
242            } catch (Exception e) {
243                System.err.println("Error while parsing KeX output file. KeXEISystem.");
244                e.printStackTrace();
245            }
246            return returnHits;
247    
248        }
249    
250        /**
251         * Return an List of TextAnnotations for the document found in the file
252         */
253        private ArrayList<TextAnnotation> parseEntitiesFromFile(String originalTextFilename) {
254            ArrayList<TextAnnotation> returnHits = new ArrayList<TextAnnotation>();
255            try {
256                /* Open the KeX output file */
257                BufferedReader br = new BufferedReader(new InputStreamReader(new FileInputStream(this.getOutputFilename())));
258    
259                /* Open the file containing the original (untagged) document texts */
260                BufferedReader br_origText = new BufferedReader(new InputStreamReader(new FileInputStream(originalTextFilename)));
261    
262                String line;
263    
264                String origDocID;
265                String origDocText;
266                String taggedDocumentID = "";
267                String taggedDocumentText;
268                // int[] sectionIDs;
269                // int[] sectionLengths;
270                // int docCount = -1;
271    
272                /*
273                 * Get the first line to prime the system. This line should contain the document ID for the first document searched for protein
274                 * names
275                 */
276                if ((line = br.readLine()) != null) {
277                    taggedDocumentID = line.substring(2);
278                }
279    
280                /* Cycle through the output file line-by-line until there are no lines remaining */
281                while ((line = br.readLine()) != null) {
282                    /* Tagged documents can span multiple lines in the KeX output file */
283                    /* Trim the docID */
284                    taggedDocumentID = taggedDocumentID.trim();
285    
286    
287                    /* This line is part or all of the document text, so grab it. */
288                    taggedDocumentText = line;
289    
290                    /* While there are more lines that start with @@, grab those as well as they are part of the same document */
291                    while ((line = br.readLine()) != null && !line.startsWith("@@")) {
292                        taggedDocumentText += (line + " ");
293                    }
294                    /*
295                     * the "line" variable now contains the document ID for the next document, or it is null indicating the end of the file has
296                     * been reached
297                     */
298    
299                    /* get the original (untagged text) from file so we can determine the span */
300                    origDocID = br_origText.readLine().substring(2).trim();
301                    origDocText = br_origText.readLine();
302    
303                    if (DEBUG) {
304                        System.out.println("TAGGED DOC ID: " + taggedDocumentID + "  ORIG DOC ID: " + origDocID);
305                    }
306    
307                    
308                    List<TextAnnotation> proteinAnnotations = this.parseHitsFromKeXOutputFile(taggedDocumentID, taggedDocumentText, origDocID,
309                            origDocText);
310                    returnHits.addAll(proteinAnnotations);
311    
312                    /* get docID for the next document */
313                    if (line != null) {
314                        taggedDocumentID = line.substring(2);
315                    }
316                    this.reset();
317                } /* End while loop over all of the lines in the output file */
318    
319                /* close file readers */
320                br.close();
321                br_origText.close();
322    
323            } catch (Exception e) {
324                System.err.println("Error running KeXEISystem...");
325                e.printStackTrace();
326            }
327            return returnHits;
328        }
329    
330        private List<TextAnnotation> parseEntitiesFromString(String originalText, String originalDocID) {
331            List<TextAnnotation> returnHits = new ArrayList<TextAnnotation>();
332            try {
333                /* Open a file reader for the KeX output file */
334                BufferedReader br = new BufferedReader(new InputStreamReader(new FileInputStream(this.getOutputFilename())));
335                String line;
336    
337                String taggedDocumentID = "";
338                String taggedDocumentText;
339    
340                if ((line = br.readLine()) != null) {
341                    /* remove any p tags that may have been put on this line -- this happens for the BioCreative corpus*/
342                    line = line.replaceAll("<p>","").replaceAll("</p>","");
343                    taggedDocumentID = line.substring(2);
344                }
345    
346                while ((line = br.readLine()) != null) {
347                    /* tagged documents are on multiple lines due to KeX processing
348                     * first line is the doc id, the rest are sentences */
349                    taggedDocumentID = taggedDocumentID.trim();
350                    
351                    /* get docText from file, remove line breaks within text */
352                    taggedDocumentText = line;
353                    while ((line = br.readLine()) != null && !line.startsWith("@@")) {
354                        taggedDocumentText += (line + " ");
355                    }
356    
357                    List<TextAnnotation> proteinAnnotations = this.parseHitsFromKeXOutputFile(taggedDocumentID, taggedDocumentText,
358                            originalDocID, originalText);
359                    if (DEBUG) {
360                        System.out.println("RETURNING " + proteinAnnotations.size() + " PROTEIN ANNOTATIONS FROM KEX OUTPUT.");
361                    }
362                    returnHits.addAll(proteinAnnotations);
363    
364                    /* The next line should be null */
365                    if (line != null) {
366                        /* There should only be one document in this file, so ERROR if the line is not null */
367                        taggedDocumentID = line.substring(2);
368                        System.err.println("WARNING in KeXEISystem. Unexpected document found during returnHitsFromString");
369                    }
370                }
371    
372                /* close file reader */
373                br.close();
374    
375            } catch (Exception e) {
376                System.err.println("Error running KeXEISystem... returnGeneHitsFromString.");
377                e.printStackTrace();
378            }
379            return returnHits;
380        }
381    
382    }
```
